# 4364 Ethicists’ Viewpoints on Face Transplant: A survey study to guide clinical practice

**DOI:** 10.1017/cts.2020.388

**Published:** 2020-07-29

**Authors:** Marissa Suchyta, Richard Sharp, Hatem Amer, Elizabeth Bradley, Samir Mardini

**Affiliations:** 1 Mayo Clinic

## Abstract

OBJECTIVES/GOALS: Face transplant can offer functional and aesthetic restoration to patients who have exhausted reconstructive options. Ethical issues in face transplant still abound, including that of patient selection. The goal of this study was to assess ethicists’ viewpoints on face transplant. METHODS/STUDY POPULATION: A large-scale online survey of attendees of the International Conference on Clinical Ethics Consultation (N = 401) was performed to assess ethicists’ opinions on issues in face transplant. Questions were asked regarding the risk-benefit ratio of immunosuppression, permissibility of face transplant for more recipient subpopulations (including children and blind patients), donor-recipient age, gender, and ethnicity mismatches, and ethics committee make-up. RESULTS/ANTICIPATED RESULTS: Among 84 respondents, 84% agreed it is permissible to perform a face transplant on an adult with no medical contraindications. The majority of respondents agreed that it is permissible to perform a face transplant on a child or blind recipient. An issue of continued concern was risk of immunosuppression. Respondents had a high threshold of permissibility for ethnic mismatches between donor and recipient, and 43% reported it is permissible to have a gender mismatch. A 10 year age difference between donor and recipient was the most commonly accepted. Questions regarding the ideal composition of a face transplant ethics committee demonstrated consensus on the roles that should be represented. DISCUSSION/SIGNIFICANCE OF IMPACT: This study provides insight into ethicists’ viewpoints on face transplant, which demonstrates a high level of permissibility towards the procedure. This may be due to the early success of face transplants and the shifting ethical issues in the field to practical aspects of the procedure. This research also provides guidance to programs regarding questions of donor and recipient selection, ethics committee composition, and offers insight into strengthening the ethical framework of the field.

